# Azov-type spits: long-term monitoring of morphodynamics and vegetation in response to changing environment

**DOI:** 10.7717/peerj.18369

**Published:** 2025-04-16

**Authors:** Oksana Tyshchenko, Volodymyr Tyshchenko, Svitlana Boychenko, Andrii Tarieiev, Vasyl Tkachenko

**Affiliations:** 1 Plant Biology Department, Taras Shevchenko National University of Kyiv, Kyiv, Ukraine; 2 Department of Preservation and Recreational Activities, State Ecological Academy of Postgraduate Education and Management, Kyiv, Ukraine; 3 Department of Fauna and Systematics of Vertebrates, I.I. Schmalhausen Institute of Zoology of National Academy of Sciences of Ukraine, Kyiv, Ukraine; 4 S. I. Subbotin Institute of Geophysics of the NAS of Ukraine, Kyiv, Ukraine; 5 Department of Environmental Studies, National University of Kyiv-Mohyla Academy, Kyiv, Ukraine; 6 Department of Forest Genetics and Forest Tree Breeding, Georg-August-University of Göttingen, Göttingen, Germany; 7 Ukrainian Botanical Society, Kyiv, Ukraine

**Keywords:** Azov-type spits, Climate change, Morphodynamics, Vegetation changes, Anthropogenic influence

## Abstract

**Background:**

Azov-type spits (ATS) are unique landforms located along the Northern coast of the Sea of Azov (NA) that have no global analogs. They play a vital role in delivering essential ecosystem services and significantly contribute to the economy of southern Ukraine. ATS are highly sensitive and dynamically responsive to environmental changes, including global and local climate changes, rising sea levels, geological shifts in the Ukrainian crystalline shield, internal shifts in the Sea of Azov and various anthropogenic influences. These factors significantly shape the ATS, influencing their capacity to accumulate biogenic material and sediments, thereby impacting vegetation cover, resilience and functioning within their ecological context.

**Methods:**

Our study on ATS morphodynamics and vegetation changes is based on a 95-year dataset that incorporates retrospective vegetation maps (1927–1929, 1934, 1996–1999), grassland releves (1995–1999) and satellite imagery (1975–2022) using specific standardized indices (NDVI, NDWI, NDMI, Thermal). We employed Earth Remote Sensing (ERS) tools due to the impracticality of field research amid the ongoing military occupation of ATS territories. Climate change vulnerability was assessed by examining surface air temperature and precipitation changes for the periods 1900–2021 and 1991–2021.

**Results:**

Meteorological data for NA shows a consistent climate change trend, including rising annual surface temperatures (1.14 ± 0.3 °C/100 years) and increased annual precipitation (98 ± 35 mm/100 years) over the last 120 years. Recent decades have witnessed intensified aridization, with up to a 15% drop in precipitation and a 0.8 °C per decade temperature increase, accompanied by increased evaporation. Our study reveals the ongoing transformation of ATS and their vegetation, primarily driven by inundation, aggravated by climate change and rising sea levels. ERS tools demonstrated their effectiveness in monitoring environmental changes under challenging circumstances, identifying general trends in the state of plant communities and validating our earlier forecasts for changes in vegetation cover. The increase in the area of halophytic meadow and marsh plant communities occurred alongside a certain decrease in their productivity, while the reduction in sandy-steppe plant community areas was accompanied by an increase in their productivity. The study provides a complex evaluation of the current anthropogenic impacts on the spits and their vegetation.

## Introduction

**Figure 1:**
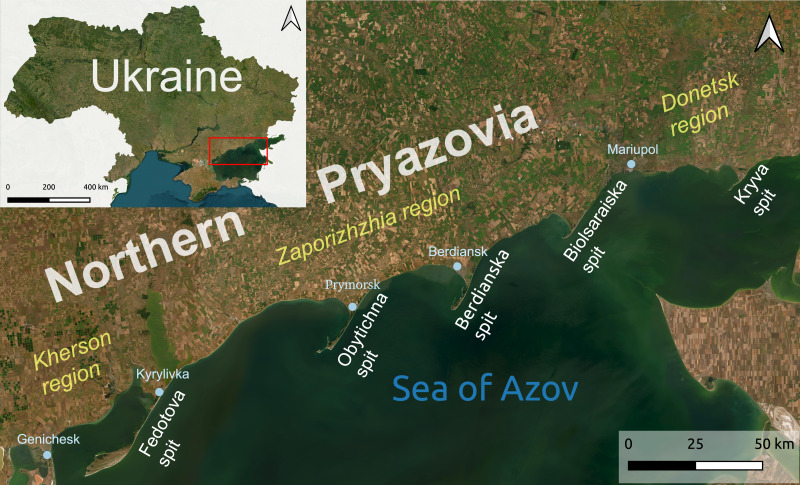
Location of the study area with ATS as the study sites. Created in QGIS 3.38.1 Grenoble using Google Satellite Hybrid Map and ESRI (Environmental Systems Research Institute).

Coastal ecosystems play a crucial role in maintaining biodiversity, delivering ecosystem services, and providing natural shoreline protection. The Azov-type spits (ATS) are located along the Northern coast of the Sea of Azov (NA), spanning from 46.2°N to 47.1°N and from 35.2°E to 38.1°E (Fig. 1). They represent unique landforms that emerged relatively recently, around the late Pleistocene-Holocene transition. ATS are classified as accumulative landforms within the globally distributed family of cuspate spits (Zenkovich, 1959; Rosen, 1975; Rosen, 1982), which represent cuspate projections of a shoreline into a body of water. These formations are thought to develop due to the reorientation of the shoreline in response to dominant wind and wave approaches, along with sediment transportation and deposition by longshore currents. In this study, we adopt Zenkovich’s (1958) definition of the term “Azov-type spits”, who coined it to specifically designate the exceptional spit system found along the NA. According to Zenkovich (1958), Zenkovich (1959) and Zenkovich (1967), ATS are accumulative, low-lying, wedge-shaped sandy-shell peninsulas that extend into the Azov Sea at a 45° angle to the shoreline, oriented northeast to southwest, featuring a broad triangular northern base, a narrow central section, and an elongated southern tip that curves southwest. With these distinctive natural features shaping their overall morphology and dynamic characteristics, these spits stand without global analogs and provide essential ecosystem services that benefit both the environment and human communities.

ATS formation is closely related to the history of the Sea of Azov’s development, fluctuations in the global sea level, and the geological history of the Ukrainian crystalline shield. The shores of the Sea of Azov are mainly composed of abrasive deposits from the Neogene and Quaternary periods. NA is fragmented by a system of tectonic faults in the basement into isolated blocks, each with its own direction of tectonic movements. The spits are associated with the subsided areas (grabens) within these blocks. ATS are accumulative formations primarily composed of mollusk shells (can constitute as much as 60–90% of the spits’ forming material) and quartz sand, with the highest elevation reaching up to three m above sea level (except Stepok island, which is embedded in Fedotova spit, and partly elevated at 5–6 m above sea level). The transportation of this material occurs through currents in the sea, which are created under the influence of prevailing winds from the northeast direction, resulting in the formation of a clockwise water circulation in the Sea of Azov (multiple sources summarized in Tyshchenko, 2006).

Typically, the deposition of the main forming material takes place on the eastern coast of each ATS. The spit gradually lengthens, so the body of the spit is oriented at an angle of about 45° to the coastline, extending deep into the sea, and oriented from the northeast to the southwest, while the end part of the spit has a curved form. Along the western shore, a shallow bay with a gentle marshy shore forms.

Until 2022, ATS ecosystems played a pivotal role in the economy of southern Ukraine, serving as resources for recreation, tourism, fishing, ports, harbors, maritime navigation, and natural wave and storm barriers. The major part of ATS territories located within five Wetlands of International Importance, four Emerald Network sites, and 22 protected areas of Ukraine’s national nature reserve fund, including three national nature parks, seven natural monuments, and 12 zakaznyks (IUCN Category IV—habitat or species management areas). Numerous plant species and communities in the ATS are subject to conservation measures, including listings in the Red Book of Ukraine (seven species), European Red List (eight species), IUCN Red List (seven species), Bern Convention Appendix I (three species), and protection of three plant communities at the state and regional levels.

ATS are dynamic structures, and their contours, condition and vegetation are constantly changing in response to the impact of various factors, which are discussed in detail in the sections “Features of Morphodynamics in ATSP for the 95-Year Period (1927–2022)” and “Environmental Effects of Human Activities in NA: Potential Impact on Spits”. The vegetation of individual ATS has been a subject of interest for many researchers since the beginning of the 20th century. However, only the studies by Popovych (1936) and Postryhan (1939) in the 1920s–30s, along with our research in the 1990s, can be considered sufficiently detailed to establish a comprehensive monitoring series. Over the past nine decades, a noteworthy increase in annual temperatures, primarily attributed to global warming, has been observed in the Sea of Azov region (Sea Level, 2023). In conjunction with shifts in climatic conditions, alterations in the hydrological regime of the Sea of Azov and human-induced changes, these transformations directly influence the composition of plant communities in ATS. This leads to changes in plant diversity and ecosystem functions, ultimately impacting the overall resilience and stability of ATS ecosystems.

The necessity for ongoing monitoring arises from a recent evaluation of the condition and vegetation within the ATS, considering significant changes over the nearly three decades since the previous study. Given the ongoing war of aggression against Ukraine and current military occupation of ATS, conducting field research is presently hindered and is anticipated to remain so in the near future, owing to ongoing demining requirements and addressing other military-related consequences. To address this challenge, our study uses Earth remote sensing (ERS) data along with various temporal climate indicators. This approach enables us to sustain long-term monitoring of morphodynamics and vegetation trends by tracking changes in selected ATS.

For this purpose, we aim to:

• continue monitoring research that was started in the 1920s and 1990s in selected ATS;
• track the most relevant climate factors influencing the transformation of ATS and their vegetation by analyzing a set of statistically processed retrospective meteorological data;
• use retrospective vegetation maps, ERS tools and retrospective satellite imagery to assess contemporary morphodynamic processes within the confines of ATS and the ongoing changes in their vegetation cover, as well as to verify our previous forecast from 2001 in the light of the changing environment.

## Materials & Methods

Within the current study, we used a combination of field methods for studying vegetation cover and methods for processing meteorological, phytosociological, cartographic and satellite remote sensing data.

### Study area and sites’ selection

The study area is located in the NA and comprises five ATS: Kryva (10 km), Bilosaraiska (14 km), Berdianska (23 km), Obytichna (30 km), and Fedotova (45 km). These spits, along with smaller non-Azov-type spits (Samsonova, Bezimenna, Shyrokynska, Liapynska), are situated in the Donetsk, Zaporizhzhia, and Kherson regions of Ukraine (Fig. 1). We selected four of the five ATS as model polygons (referred later in text as Azov-type spits polygons (ATSP)), namely Kryva, Bilosaraiska, Berdianska and Obytichna. These spits comprehensively represent specific ATS and have the best-documented materials from retrospective studies, as discussed later in the ‘Materials & Methods’. The transliteration of Ukrainian geographic names in this text has been done using the National System of Romanization of Ukrainian language (Romanization system in Ukraine, 2012).

### Different temporal climate indicators of NA

The analysis explores the causes of changes in vegetation cover on ATS by examining meteorological data from southern Ukrainian stations spanning 1900 to 2021. Using empirical data from Henichesk, Mariupol and Melitopol meteorological stations, the research incorporates averaged monthly surface air temperature and precipitation values (Open Data, 2022; CGO, 2021; Climate Explorer, 2023). Additionally, the study incorporates climatic norms (T_nrm_ and P_nrm_) for stations Berdiansk, Botieve, Henichesk, Mariupol, and Melitopol, covering 1961–1990 (The climate Cadastre of Ukraine, 2005) and 1991–2021 (CGO, 2021). Despite recent data unavailability due to the Russian war against Ukraine and station destruction in an occupied zone since February 2022, the analysis compares long-term trends from 1900–2021 with recent changes from 1991–2020. Linear regression and the Mann–Kendall test assess trends, revealing significant climate shifts. The statistical analyses and graphical representations were conducted using MS Excel and XLSTAT software. More details are provided in the Supplemental Information 1.

### Retrospective vegetation maps

The initial vegetation status of ATS was assessed using vegetation maps created by Postryhan (1939) and Popovych (1936) in the 1920s–1930s, along with large-scale vegetation maps developed in the 1990s (Tyshchenko, 2006). This collection forms the overall cartographic database, serving as a foundational resource for ongoing monitoring of natural processes in the NA region. It facilitates the evaluation of long-term changes in spatial structure, dynamics, and quantitative alterations in the vegetation cover of the spits. More details are provided in the Supplemental Information 2.

### ERS tools

ERS tools were used to analyze the morphodynamics and long-term vegetation changes in ATSP, considering various indicators. The EO Browser web platform (https://apps.sentinel-hub.com/eo-browser/) facilitated the analysis of satellite images from Sentinel-2 L2A, Landsat 4–5 TM, Landsat 1–5 MSS L1, and MODIS sources spanning 1975 to 2022. Sentinel Hub tools enabled a comprehensive examination using specific indices NDVI, NDWI, NDMI and Thermal. More details are provided in the Supplemental Information 3.

## Results

### Features of climate change in NA for the 121-year period (1900–2021)

The Northern coast of the Sea of Azov has a moderately continental climate, influenced by maritime breeze circulation, and exhibits pronounced seasonality (Babychev & Marynych, 1989; Lipinskyi, Dyachuk & Babichenko, 2003; Ilyin et al., 2009). The atmospheric circulation, which carries marine air masses from the Atlantic and Arctic seas to the region of the Sea of Azov and its coasts, as well as continental masses from various regions of Eurasia, plays a dominant role in shaping the temperature regime. The region’s climate is characterized by warm, dry summers that occasionally experience heavy rains, and relatively mild winters. The average annual temperature ranges from about +9.1 to +10.5 °C. Summers here are warm and arid, with temperatures that can rise to +30 °C and more, although they usually fluctuate between +20 to +24 °C. Winters are mild, with an average January temperature ranging from −1 °C to −3 °C, but temperatures can sometimes drop to −10 °C. The annual precipitation is about 350–500 mm, with the majority falling during the warm period of the year (Lipinskyi, Dyachuk & Babichenko, 2003).

**Figure 2:**
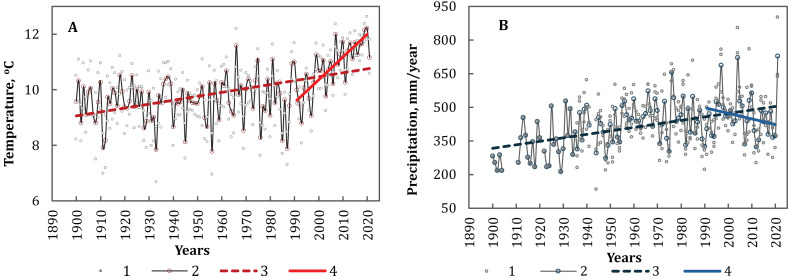
The annual average temperature (A) and the annual amount of precipitation (B) in the NA. 1—empirical data from the meteorological station, 2—average values of the NA, 3—linear trend for the period 1900-2021, 4—linear trend for the period 1991-2020. Statistically significant trends (*p* < 0.05).

According to the analysis of meteorological data of stations located in regions of NA with long–time series (Henichesk, Mariupol, Melitopol), the average annual temperature was 9.9 ± 1.0 °C, and the average annual amount of precipitation was 410 ± 96 mm/year for the period 1900–2021. Consistent with regional trends, certain climatic changes have been observed in the Azov region (Dyakov, Ivanov & Gorbach, 2002; Voskresenskaya & Naumova, 2006). The average annual temperature has increased by 1.14 ± 0.30 °C per 100 years, while the annual amount of precipitation has increased by 98 ± 35 mm per 100 years (Figs. 2A, 2B).

Studies have shown that in Ukraine, including Crimea (Lipinskyi, Dyachuk & Babichenko, 2003; Ilyin et al., 2009; Boychenko, 2008; Boychenko & Kuchma, 2022), the pace of climate change is in line with global trends (IPCC, 2013; IPCC, 2021). Specifically, in the southern region of Ukraine, there has been an increase in temperature of more than 1.0–1.2 °C per 100 years, and a slight increase in the amount of precipitation from the end of the 19th century to the early 21st century (Karamushka et al., 2022).

Moreover, the trend of rapid temperature increase has been observed both globally and regionally over the past 30 years (Karamushka et al., 2022; IPCC, 2013). On the territory of the NA, the average annual temperature has already reached 10.8 ± 0.9 °C, and a trend of increasing temperatures by 0.80 ± 0.07  °C per decade has been established for the period 1991–2020. While temperatures have been rising in the NA, there has concurrently been a decrease in annual precipitation for this period. The average annual amount of precipitation was 454 ± 105 mm per year, and the region has experienced an average reduction of −25 ± 9 mm per decade for the period 1991–2020.

It should be noted that in the 20th century, there was a trend towards an increase in precipitation by 10–15% in the southern, southwestern, and south-eastern regions of Ukraine (Boychenko, 2008; Voloshchuk & Boychenko, 2003). Additionally, for the period 1991–2020, an increase of approximately 10% was also established (Karamushka et al., 2022). However, the analysis of data from coastal meteorological stations of the Northern Azov Sea showed a trend towards a decrease in precipitation of up to 15%. Similar trends were also established for the Crimean Peninsula (Boychenko & Kuchma, 2022; Boychenko, Kuchma & Khlobystov, 2022). We assume that this is related to the influence of anticyclones bringing dry air from the south-eastern regions of Asia to NA and the Crimean Peninsula.

**Figure 3:**
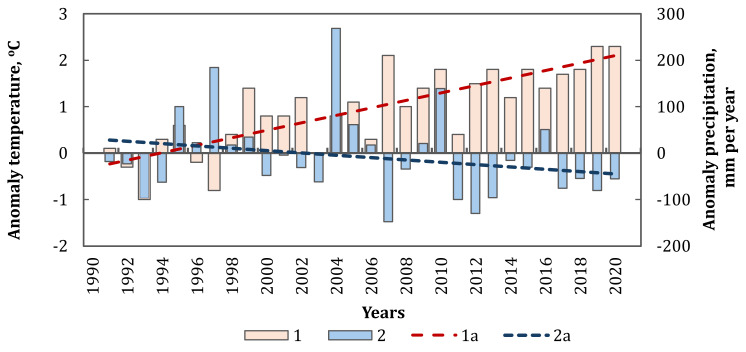
Comparison of the anomalies in the annual average temperature (1) and the amount of precipitation (2) in the NA region (1a and 2a are linear trends, respectively) for the period 1991–2020. Statistically significant trends (*p* < 0.05).

This is well illustrated by the figure, which shows a comparison of the anomalies in the annual average temperature and the amount of precipitation in the NA region for the period 1991–2020 (Fig. 3). Parameters of anomalies were counted from the climatic norm of meteorological parameters for the period 1961–1990, namely T_nrm_ = 9.9 °C, P_nrm_ = 445 mm per year. Also, a comparison of climatic norms of annual average temperature and precipitation, along with their trends, was made for the respective meteorological stations of the NA region. The data was collected for two periods: 1961–1990 and 1991–2020, and the results are presented in Table 1.

**Table 1:** Comparison of climatic norms of annual average temperature and precipitation, along with their trends, for the respective meteorological stations in the NA region for the periods 1961–1990 and 1991–2020.

Station	Temperature			Precipitation		
	1961–1990	1991–2020		1961–1990	1991–2020	
	Tnrm ±*σ*, °C	Tnrm ±*σ*, °C	Ttrn, °C per 100 years	Pnrm ±*σ*, mm per year	Pnrm ±*σ*, mm per year	Ptrn, mm per 10 years
Berdiansk	10.2 ± 0.9	11.2 ± 0.9	0.83	467 ± 96	467 ± 91	−22
Botieve	9.6 ± 0.9	10.6 ± 0.9	0.76	431 ± 82	431 ± 117	−23
Henichesk	10.5 ± 0.9	11.2 ± 0.8	0.68	398 ± 96	398 ± 94	−31
Mariupol	9.2 ± 0.9	10.3 ± 0.9	0.84	498 ± 104	498 ± 117	−37
Melitopol	9.9 ± 0.9	10.9 ± 0.9	0.83	475 ± 91	475 ± 102	−12

**Figure 4:**
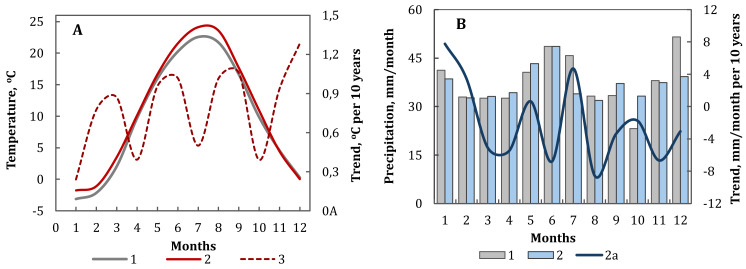
Seasonal variation of temperature (A) and precipitation (B) of the NA region. Statistically significant trends (*p* < 0.05). The values of the climatic norm for the whole region for the periods 1961–1990 (1) and 1991–2020 (2) (scale on the left), the values of linear trend (2a) for the period 1991–2020 (scale on the right).

Trends in seasonal changes of climate conditions varied depending on the period. Thus, during the 20th century, there were significant trends of temperature increase during the cold period of the year (Lipinskyi, Dyachuk & Babichenko, 2003), while in recent decades, an intensive increase in temperature has been observed throughout the year (Fig. 4A) (Karamushka et al., 2022). There has been a warming trend of 0.3 to 1.3 °C per decade for all months of the year during the period 1991–2020. Concurrently, there has been a general decrease in precipitation in March, April, and June, and from August to December by −1.8 to −8.6 mm/month per decade. However, slight increases in precipitation have been observed in January, February, May, and July by 0.6 to 7.8 mm/month per decade (Fig. 4B). It should be noted that over the last 20–30 years, there has been a significant increase in the average annual temperature in the climatic–landscape zone of the Azov Sea region. This increase is manifested by a higher frequency of periods with abnormally high winter temperatures and a slight increase in the annual amount of atmospheric precipitation (Boychenko, 2008; Dyakov, Ivanov & Gorbach, 2002; Voskresenskaya & Naumova, 2006).

New temperature records (temperatures above 30 °C) have been repeatedly registered in various regions of Europe, including Ukraine, over the past decades (Karamushka et al., 2022; Sedlmeier, Feldmann & Schädler, 2018; Bordi, Fraedrich & Sutera, 2009; Efimov et al., 2015; Koffi & Koffi, 2008). The frequency and duration of summer heat have increased, and droughts occur more often and cover larger territories (Sedlmeier, Feldmann & Schädler, 2018; Bordi, Fraedrich & Sutera, 2009; Boychenko & Kuchma, 2022). Thus, over the past few decades, there has been an intensification of aridization in the climatic conditions of NA (Boychenko & Kuchma, 2022). This is characterized by a significant decrease in precipitation by up to 15% and an increase in surface temperature by 0.80 °C per decade, accompanied by an intensification of the evaporation process.

For possible climate change scenarios in the southern region, global temperature change scenarios RCP4.5 and RCP8.5 were considered until the end of the 21st century (Krakovska et al., 2017; Krakovska et al., 2018), as well as semi–empirical scenarios for Ukraine (Scenario 1 and Scenario 2) (Boychenko, 2008; Voloshchuk & Boychenko, 2003), and climate change trends in the region, starting from the end of the 19th century and into the early 21st century. Thus, the climate scenarios for this climatic zone are as follows (scenarios have a baseline from 1900): optimistic scenario (RCP4.5) –ΔT2050 ≈ 1.4 ± 0.3 °C; pessimistic scenario (RCP8.5) –ΔT2050 ≈ 2.2 ± 0.3 °C.

### Meteorological causes of sea level changes (variability of wind speed and direction, waves, surge processes)

The relief-forming factor in the development of the coastal zone and spits of the northern coast of the Sea of Azov is short-term, non-periodic fluctuations in sea level of a synoptic nature (Ivanov et al., 2008; Ilyin et al., 2009; Ivanov, Cherkesov & Shulga, 2012).

According to the data given in (Ivanov et al., 2008; Ilyin et al., 2009), the average annual values of surface wind speed in the coastal zone of *NA* for the period 1936–2007 vary from 4.4 to 6.0 m/s. In the Mysove area, wind speeds reach 6.1 ± .4 m/s. There is a tendency for speeds to decrease by 0.15–0.35 m/s per 10 years at most compass points. In the seasonal course, wind speed has a pronounced maximum in November–March (from 5.0 to 6.9 m/s) and a minimum in the summer months (from 3.8 to 5.3 m/s) (Ilyin et al., 2009). Maximum wind speeds in most of the coastal zone of NA reach 29 ± 5 m/s. In the Azov Sea basin, the east wind direction is predominant in the cold season with a frequency of 23–36% (19–22% per year) (Ilyin et al., 2009). North-east winds are observed in winter with a frequency of 17–24% (13–23% per year). Moreover, storm winds with a speed of 15 m/s in these directions have a frequency of 1–3%. There is a slight decrease in the frequency of strong east and north-east winds in the winter season (Genichesk, Berdiansk) and, conversely, an increase in the spring in the east (Berdiansk, Mysove). In the southern direction, the frequency of winds is 5–10% with a tendency to increase in the warm season. The frequency of north winds is 9–17% with no tendency to change. West and north-west winds have a frequency of 15–18%, with insignificant changes (Fomin et al., 2012). In the seasonal course, the activation of these directions is recorded in the summer, while in the fall, on the contrary, a slight decrease is observed (Dyakov & Fomin, 2002; Ivanov et al., 2008; Ilyin et al., 2009).

The waves in the Sea of Azov are influenced by wind conditions above the sea, the bottom relief, the configuration of the coastline, and the extent of sea ice coverage during the cold season (Ivanov et al., 2008; Ilyin et al., 2009; Vykhovanets & Stoyan, 2012). The waves primarily originate from the eastern, northeastern, western, and southwestern directions, which align with the wind regime of the sea. In the coastal zone and near the spits, mild waves prevail; in 92 ± 3% of cases, the wave height does not exceed 0.5–0.7 m, in 5 ± 3% of cases, there is calm, and the remainder consists of waves with a maximum height of up to 2–3 m (Berdiansk, Mariupol, Mysove). Consequently, coastal erosion occurs along the northern coast and in the southeastern regions of the sea (Ilyin et al., 2009). Another notable characteristic of the Sea of Azov’s wave regime is the virtual absence of swell waves. A feature of the storm wave regime in autumn is the emergence of Mediterranean cyclones in November, which are accompanied by strong storms from southern directions. For instance, in November 1992 and 2007, the impact of waves and surges led to the destruction of coastal infrastructure and the erosion of the sea spits. It has been noted (Shulga, 2011; Ivanov, Cherkesov & Shulga, 2012; Ilyin et al., 2009) that in recent decades, there has been a trend of low-intensity waves and a decrease in the frequency of storm waves.

In certain synoptic situations, strong, prolonged winds with speeds of 15 m/s or more can cause significant rises or falls in sea level in specific areas (Dyakov, Ivanov & Gorbach, 2002). Depending on the atmospheric processes occurring over the waters of the Northern Azov, these surge phenomena can last from several hours to several days. In the region, the average number of days with storm winds is 29 ± 5 days per year (Dyakov & Fomin, 2002). Surge fluctuations in the level of the Sea of Azov have the form of a seiche with one nodal line passing approximately through the center of the sea. The eastern coast of the sea and the Taganrog Bay are most frequently affected by catastrophic surges, occurring in 48% of all cases, with surges occurring in 60% of instances (Dyakov & Fomin, 2002; Ivanov, Cherkesov & Shulga, 2012). The amplitude of wind-driven fluctuations in sea level can sometimes reach 2.5 ± 0.5 m. It is believed that the significant frequency of storm winds from the eastern compass directions during the cold period of the year can lead to a tilt in the sea level surface, with a gradient from the northeast to the southwest of the sea due to wind-driven processes (Ilyin et al., 2009; Shulga, 2011). As a result, the difference in sea level between the southwest coast of the Sea of Azov and the Taganrog Bay can average 12–14 cm (Ilyin, 2011). The greatest amplitudes of sea level fluctuations are observed in the Taganrog Bay (609 cm) and Genichesk (412 cm), with the smallest amplitudes in the Kerch Strait (193 cm) (Dyakov & Fomin, 2002). However, average annual sea level fluctuations do not significantly depend on wind-driven processes. Therefore, based on long-term changes in sea level and considering the current tectonic situation, the recorded levels on the northern coast can be indicative of the overall changes in the level of the entire Sea of Azov (Goryachkin, 2008; Ilyin et al., 2009).

Ice conditions in the Sea of Azov have a peculiarity of ice formation even in years with mild winters and the possibility of intensive drift of ice fields. However, due to warming in the last thirty years, the appearance of ice has shifted to 8–10 days later, and the sea has begun to clear of ice earlier by 10–17 days (Ilyin et al., 2009).

### Features of morphodynamics in ATSP for the 95-year period (1927–2022)

The coastal strip of NA is characterized by high dynamics of natural processes which are associated with the dynamic processes of the Sea of Azov. In the case of the ATS, the condition and changes of their coasts are primarily influenced by abrasion-accumulation processes, fluctuations in sea level, alterations in wind-wave regime characteristics, and variations in the volumes of biogenic material that form their bodies. Among these factors, the biogenic factor plays a pivotal role in maintaining the balance of these accumulation forms. In recent decades, due to the increasing impact of human activities on natural systems, there have been intensifications of various geomorphological processes in the area of the ATS location and adjacent continental slopes. These processes include intensified erosion, littoral zone washouts, increased occurrence of landslides, coastal areas inundation. Significant changes in coastal sediment flows along the native coastline of the NA are caused by numerous coastal defense and port structures (Zinchenko, 2016), which notably reduce the rate of abrasion and the amount of detrital material reaching the ATS coastal zone. Therefore, it becomes crucial to assess the contemporary changes in the morphology of the ATS coasts, and to identify the trends and mechanisms of these changes.

The morphodynamic features of ATSP are analyzed by comparing data from the period 1927 to 1998 (Tyshchenko, 2006) with more recent satellite imagery spanning from 1975 to 2022. This comprehensive analysis provides insights into morphodynamic changes of ATSP over a 95-year timeframe (Supplemental Information 4).

Our research has revealed that Berdianska and Obytichna spits, situated farther west and extending further into the sea, undergo more substantial erosion. The primary driver behind most of the alterations in the coastal spits of NA is associated with the rise in global sea levels, a consequence of the ongoing global warming process. However, we do not discount the possibility of local tectonic subsidence or a combination of these processes, along with the decline in shell material production due to changes in sea salinity and pollution, contributing to these changes.

### ATSP vegetation cover changes for the 95-year period (1927–2022)

The vegetation on ATSP is primarily composed of an intrazonal type of vegetation, which consists of littoral, psammophytic-steppe, halophytic-meadow, halophytic, marsh and aquatic vegetation. The development of plant communities in this region typically results in the formation of zonal steppe phytocenoses or structurally similar assemblages. However, the continuous influence of the sea, along with edaphic, hydrochemical, and climatic factors, imposes limitations on the growth and establishment of plant communities at the intrazonal level of vegetation.

Re-inventory surveys on ATSP revealed significant changes over a 70-year period (from 1927–1934 to 1996–1999). These changes were particularly notable in halophytic vegetation of the *Therosalicornietea* Tx. in Tx. et Oberd. 1958 and *Kalidietea foliati* Mirkin et al. ex Rukhlenko 2012 classes, which decreased in area by 13 times. The most substantial decline was observed on Kryva spit, where halophyte vegetation cover decreased by over 80 times from 30% in 1929 to 0.4% in 1998. Similar declines were seen on Bilosaraiska spit, decreasing from 30% to less than 1%, and on Berdianska spit, reducing from 24% to 0.2%. Obitychna spit experienced a decrease from 22% in 1934 to 10% in 1996.

During this period, the area occupied by halophytic meadow plant communities of the *Festuco-Puccinellietea* Soó ex Vicherek 1973 and *Juncetea maritimi* Br.-Bl. in Br.-Bl., Roussine et Nègre 1952 classes nearly tripled. On Kryva spit, it grew from approximately 7% in 1929 to over 20% in 1998, marking a threefold increase. On Bilosaraiska spit, it expanded sixfold, from 7% in 1927-29 to almost 45% in 1998. Berdianska spit saw a slight increase of 1% (from 8% in 1929 to 9% in 1998). Meanwhile, Obytichna spit experienced a fourfold increase, from 9% in 1934 to 35% in 1996.

Data from the comparison of vegetation maps indicated that between 1927 and 1998, waterlogged areas on the spits expanded almost twofold, including an increase in internal water bodies. On Kryva spit, internal water bodies increased from 12% in 1929 to 23% in 1998; on Bilosaraiska spit—from 3% in 1927-29 to 14% in 1998; on Berdianska spit—from 11% in 1929 to 21% in 1998; on Obitychna spit—from 26% in 1934 to 36% in 1996.

The area covered by marsh vegetation of *Phragmito-Magnocaricetea* Klika in Klika et Novák 1941 and *Bolboschoenetea maritimi* Vicherek et Tx. ex Tx. et Hülbusch 1971 classes nearly doubled. On Kryva spit, it increased from 14% in 1929 to 23% in 1998; on Bilosaraiska spit - from 5% in 1927-29 to 9% in 1998; on Berdianska spit - from 6% in 1929 to 18% in 1998, while Obitychna spit experienced a slight decline from 20% in 1934 to 17% in 1996.

There was a substantial reduction in the total area of *Festucetea vaginata* e Soó ex Vicherek 1972 psammophytic-steppe communities, decreasing sixfold overall. On Kryva spit, it declined nearly 20 times, from 16% in 1929 to 0.8% in 1998; on Bilosaraiska spit, it decreased fivefold, from 15% in 1927-29 to 3% in 1998; on Obytichna spit, it halved, from 16% in 1934 to 8% in 1996.

The littoral zone plant communities of the *Ammophiletea* Br.-Bl. et Tx. ex Westhoff et al. 1946 class saw a twofold decrease in their total area. On Kryva spit, they decreased by almost three times, from 13% in 1929 to 5% in 1998. On Bilosaraiska spit—fivefold, from 10% in 1927-29 to 2% in 1998. On Berdianska spit—nearly threefold, from 13% of the total area in 1929 to 5% in 1998. On Obytichna spit, the decrease was weakly pronounced, from 14.5% in 1934 to 10.3% in 1996.

**Figure 5:**
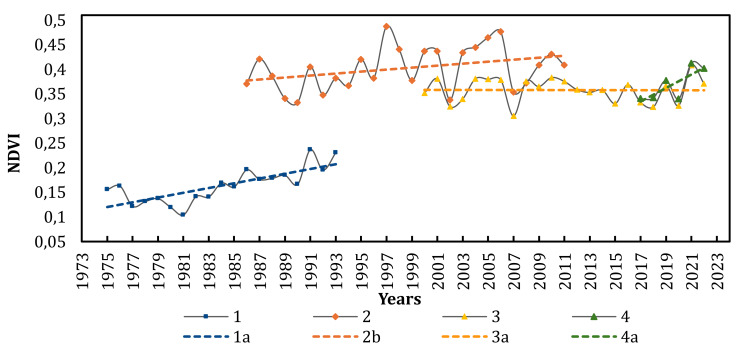
Dynamics of NDVI across the territories of ATSP from 1975 to 2022. 1—Landsat 1-5 MSS, 2—Landsat 4-5 TM, 3—MODIS, 4—entinel-2 L2A, 1a-4a—linear trends, respectively.

The NDVI analysis from 1975 to 2022 revealed a consistent increase across ATSP territories (Fig. 5, Table 2), indicating higher vegetation cover density, potentially linked to improved moisture conditions for plants and possible alterations in the dominance of plant communities.

The most intense growth in NDVI occurred during the period from 1981 to 1997. The highest increase in vegetation index was observed on Kryva spit (over 60%, Table 2).

**Figure 6:**
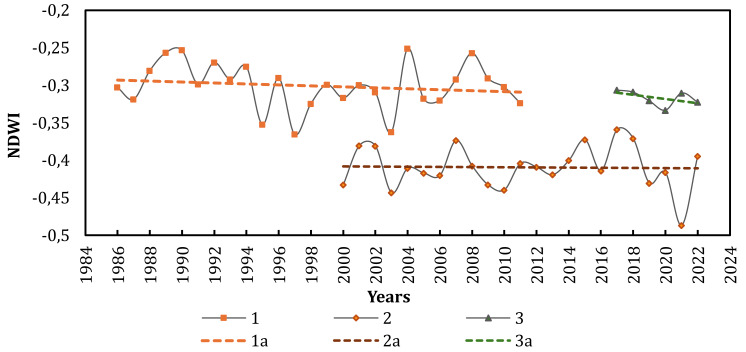
Dynamics of NDWI across the territories of ATSP from 1986 to 2022. 1—Landsat 4-5 TM, 2—MODIS, 3—Sentinel-2 L2A, 1a-3a—linear trends, respectively.

To assess changes in inland water body areas within the spits, we conducted an analysis of multi-year variations in the NDWI, which is particularly effective for mapping water features. We specifically selected areas with inland water bodies on each of the ATSP for this analysis, using satellite images taken in the month of August. For the temporal analysis of NDWI changes in the territory of all spits, data from Landsat 4–5 TM (1986–2011), MODIS (2000–2022), Sentinel-2 L2A (2017–2022) were used. The findings of the analysis revealed that, cumulatively from 1986 to 2022, NDWI decreased across the territories of the ATSP. In simpler terms, the areas of inland water bodies decreased. The most significant cumulative decrease in NDWI occurred in the territories of Kryva and Bilosaraiska spits (Fig. 6, Table 3).

**Table 2:** Changes in NDVI from 1975 to 2022 for the ATSP, %.

ATSP			
Kryva	Bilosaraiska	Berdianska	Obytichna
+60,85	+31,84	+31,05	+40,41

**Table 3:** Changes in NDWI on the ATSP from 1986 to 2022 (based on the trend regression line).

Source of images, period	Trend of changes in NDWI on the ATSP, %
Landsat 4–5 TM, 1986-2011	−4,85
MODIS, 2000–2022	−2,0
Sentinel-2 L2A, 2017–2022	−7,5

**Figure 7:**
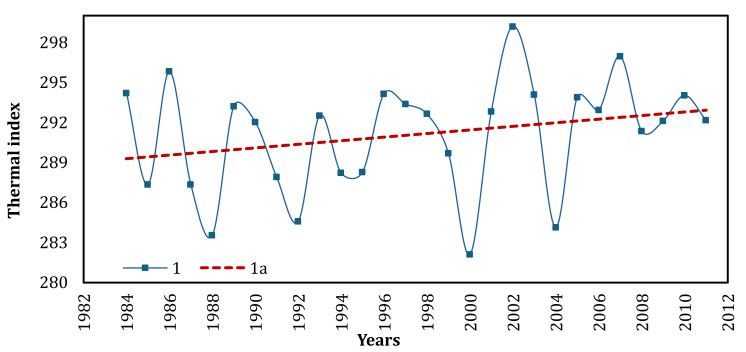
Surface temperatures on the ATSP according to Thermal sensor (Landsat 4-5, *n* = 757) satellite image data from 1984 to 2011. 1—surface temperature, 1a—linear trend.

The decrease in the area of inland water bodies within the spits is consistent with the long-term climate change trends in the region (Fig. 2), specifically the rise in average annual temperature and the reduction in annual precipitation over the last 30 years. The prolonged trend of increasing surface temperatures on the spits is additionally supported by the examination of satellite images using a thermal sensor (Landsat 4–5—Thermal TM Band 6, 1984–2011, *n* = 757, +1.4%) (Fig. 7).

In certain periods, there was an increase in the area of water bodies, likely attributable to intensified storm wave phenomena. It’s worth noting that the dynamics of changes in water body areas on Obytichna and Berdianska spits are not indicative, as they are the most vulnerable to runoff phenomena and storms.

**Figure 8:**
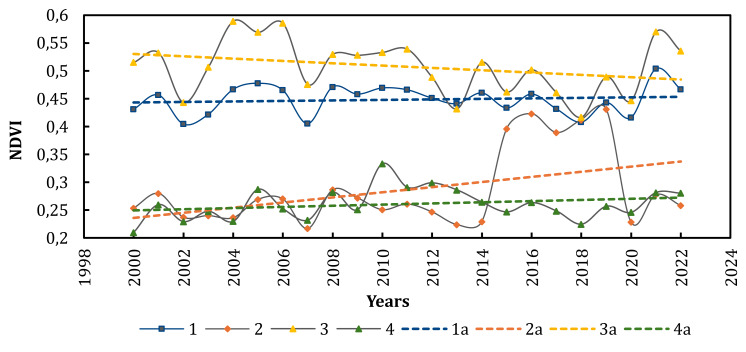
Multi-year variations in NDVI for the main types of vegetation on the ATSP. 1—Marsh, 2—Halophytic, 3—Halophytic-meadow, 4—Psammophytic-steppe , 1a-3a—linear trends, respectively.

To understand the trend of changes in the dominance of plant community types on the Azov Sea spits from 2000 to 2022, we conducted an analysis of multi-year variations in NDVI for four main, tentatively outlined types of vegetation on the ATSP: marsh plant communities (*Phragmito-Magnocaricetea* and *Bolboschoenetea maritimi* classes), halophytic-meadow (*Festuco-Puccinellietea* and *Juncetea maritimi* classes), halophytic (*Therosalicornietea* and *Kalidietea foliati* classes), and psammophytic-steppe (including littoral) vegetation (*Festucetea vaginatae* and *Ammophiletea* classes) (Fig. 8, Table 4).

**Table 4:** Rates of multi-year index changes for the main types of vegetation on the ATSP.

Plant communities	Rates of index changes, %		
	NDVI 2000–2022	NDVI 2017–2022	NDMI 2016–2022
Marsh	+4,5	+18,6	+233
Halophytic-meadow	−7,7	+69,7	+91,7
Halophytic	+19,3	+18,5	+14,9
Psammophytic-steppe	+12,5	+21,2	+81,9

The delineation of areas dominated by specific vegetation types for satellite image analysis was based on data from original large-scale vegetation maps of the spits (1996-1999). In particular, the identified areas include marsh plant communities on the territory of Berdianska and Bilosaraiska spits (covering an area of 9.78 km^2^), halophytic-meadow plant communities on the territory of Bilosaraiska spit (covering an area of 7.39 km^2^), halophytic plant communities on the territory of Obytichna and Kryva spits (covering an area of 5.71 km^2^), and psammophytic-steppe plant communities on the territory of Samsonova and Bezimenna spits (taken for analysis additionally, covering an area of 0.64 km^2^).

We used MODIS (2000–2022) and Sentinel-2 L2A (2017–2022) data for temporal analysis of 4 types of vegetation. Despite the fact that the areas with halophyte-meadow vegetation have the highest NDVI level, the vegetation index for the studied period showed a downward trend (−7.7%). A significant increase in NDVI for this type of vegetation was only observed from 2018 to 2022 (+69.7%). This trend occurred against the backdrop of increased plant moisture availability over the past six years (NDMI +91.7%).

The vegetation index for areas with marsh vegetation on the spits experienced a slight increase (+4.5%) over the study period. This happened amidst a minor reduction in the area of inland water bodies (NDWI −4.3%) and a significant increase in plant moisture availability from 2016 to 2022 (NDMI +233%). Evidently, this is due to increased inundation of areas dominated by aquatic and marsh vegetation without a substantial increase in the total area of water bodies.

The vegetation index for psammophytic-steppe vegetation showed a slight overall increase (+12.5%) over the study period with a significant amplitude of fluctuations (percentage change of 48.1%). Over the last six years, there has been an acceleration in NDVI growth (+21.2%) and an increase in plant moisture availability (NDMI +81.9%).

## Discussion

### Response of the vegetation cover of ATSP to changes in their natural environment

The current changes in vegetation cover in ATSP result from various factors, including regional and global drivers. Long-term shifts in plant community composition on ATSP are directly linked to alterations in climatic parameters and other environmental factors. Examined meteorological data indicates a response of NA’s climatic conditions to global warming. Additionally, alterations in the Sea of Azov ecosystem, influenced by anthropogenic and natural factors, significantly impact ATSP. The contemporary Holocene tectonic subsidence of the sea bottom in the southern slopes of the Ukrainian crystalline shield, where ATSP are situated, is entirely natural (multiple sources summarized in Tyshchenko, 2006; Yesipovich et al., 2020). Global sea level rise by 98 mm during the period 1993–2023, primarily caused by melting ice sheets and seawater expansion due to global warming (Sea Level, 2023; NASA Sea Level Change Portal, 2023), affects the Sea of Azov similarly. Its water level has increased over the past century at a rate similar to that of the global sea level. According to climate projection scenarios (RCP4.5 –(ΔT∼2.0 °C)), it is expected that by the year 2050, the Sea of Azov’s level will rise by approximately 25–35 cm (Boychenko et al., 2016). The majority of changes in ATSP vegetation cover can be attributed to rising sea levels, a consequence of contemporary global warming. Elevated sea levels intensify processes such as abrasion, erosion, and flooding, impacting ATSP landscapes. However, we cannot exclude the possibility of local tectonic subsidence or a combination of these processes, along with changes in sedimentation and balance processes within the ecosystem of the Sea of Azov.

ATSP vegetation is shifting towards more inundated conditions, with hydro-, hygro-, and mesophytic plant communities playing a greater role, resulting in significant alterations in their physiognomic features. Similar trends have been observed in other regions of the steppe zone of Ukraine (Tkachenko, Tyshchenko & Boychenko, 2019). The increase in halophytic meadow and marsh plant communities, and the notable decrease in the overall area of littoral and sand-steppe plant communities, signifies a long-term transition towards heightened waterlogging in most plant communities. This transformation is mainly driven by inundation due to complex drivers, detailed discussed in Supplemental Information 5. This conclusion, initially drawn over 20 years ago (Tyshchenko, 2006), partially reaffirmed now and suggests that forecasted alterations in ATSP vegetation are being realized and are expected to intensify further in the future. However, the intensification of climate change over the past two decades and the analysis of NDVI dynamics introduce certain adjustments to our forecast. The increase in vegetation productivity against the backdrop of ongoing aridization may indicate plants compensating for the lack of soil moisture from atmospheric precipitation. This could be attributed to elevated groundwater levels resulting from continuous inundation due to tectonic subsidence of the spits and sea level rise. This may also explain the decrease in productivity of halophytic meadow vegetation areas and the increase in productivity of psammophytic-steppe vegetation areas, as sandy-shell soils under these conditions are less saline and have better water supply compared to sod, sod-gley, and meadow-bog soils. The reduction in the area of inland water bodies is associated with increased evaporation rates and the overgrowth of water-released areas by marsh and halophytic vegetation. This is confirmed by the intensification of vegetation in these plant communities over the last 20 years. The questions regarding future changes in ATSP phytosystems can be further investigated through subsequent monitoring initiatives.

### Environmental effects of human activities in NA: potential impact on spits

Analysis of the situation in NA confirms that ongoing anthropogenic activities are the primary driver of the current transformation of the ATS and their vegetation. The increasing transformation of areas by human activity, coupled with climate changes and changes in the Sea of Azov ecosystem, poses a threat to the conservation and recreational potential of ATS. The contemporary anthropogenic impact on ATS territories can be divided into two periods: before the Russian war and during the war occupation. Before the war, the ATS served as vital recreational and wellness resources within Ukraine’s tourism system, particularly in the Pryazovian recreational region. These spits boasted diverse natural attributes, including a marine climate, mineral-rich waters, mud and silt, and varied ecosystems, enhancing the well-being of residents and visitors alike. However, excessive recreational activities in some areas led to significant landscape alterations (Tyshchenko, 2006).

The topography of certain areas on ATS has significantly changed due to various anthropogenic features, such as dams, roads, drainage channels, fish ponds, artificial elevations for developed areas on ATS, former quarries after sand extraction and coastal protective structures. Following adverse changes in the Sea of Azov ecosystem and the decline of fishing, related infrastructures like fleets and canneries became obsolete. Agricultural efforts failed because these areas could not develop fertile soils due to atypical soil-forming processes, resulting in nutrient-poor unique azonal soils primarily rich in sulfate-chloride salts. Traditional activities were replaced by construction and infrastructure development, but flooding hindered further progress. Excessive construction, waste, unregulated recreational activities and afforestation degraded the environment, leading to loss of biodiversity and wetland qualities. Some buildings, particularly on Berdianska, Bilosaraiska, and Fedotova spits, were constructed in flood-prone areas amidst dense reed thickets or on narrow elevated coastal strips within swampy regions, requiring periodic water removal.

The Sea of Azov’s isolation from the World Ocean makes it highly sensitive to continental runoff and atmospheric processes, impacting the regime and bioproductivity levels. ATS are particularly sensitive to changes in the Sea of Azov’s ecosystem, notably abnormal fluctuations in salinity. Before the regulation of the Don River (filling of the Tsimlyansk Reservoir in 1952) the sea’s salinity averaged 10–12‰ (Kosarev, Kostianoy & Shiganova, 2008), reaching 13.7% by the 1980s (Matishov et al., 2008) and, in some areas, 17.4% (multiple sources summarized in Tyshchenko, 2006). At the beginning of the 21st century, it returned to 10–11% (Anistratenko, Khaliman & Anistratenko, 2011), but during 2015–2022 reported local measurements exceeding 15‰ (Shukalo, Shulga & Suslin, 2023). Contemporary salinity fluctuations are linked to anthropogenic alterations in river runoff, especially from dams and reservoirs in the Kuban and Don river basins. These are exacerbated by climate change and extreme Black Sea water advections, affecting aquatic ecosystem productivity. Further salinity increases, water consumption, biogenic material accumulation in reservoirs, and the reduction of the trophic value of runoff waters will intensify ecosystem transformations in the Sea of Azov.

The aforementioned transformations, including illegal sand-shell extraction, plowing, drilling platform and pipeline construction, Crimean Bridge construction, industrial development, and industrial effluents containing hazardous pollutants (such as detergents, pesticides, heavy metals, phenols, petroleum derivatives, phosphorus organic substances, *etc*.), oil and oil product pollution, and nutrient pollution from extensive fertilizer use in agriculture, result in algal blooms and formation of hypoxic zones. These cause fish kills, loss of fishing potential in the Azov Sea, deterioration of the sanitary-epidemiological state, and degradation of recreational resources. Critical outbreaks of invasive fauna disrupt natural feeding processes and sediment balance, leading to the loss of viability of filter-feeding mollusks, including *Cerastoderma glaucum* Bruguière, 1789 and other benthic fauna species, which are primary biogenic component producers for ATS. Diminished mollusk populations reduce the spits’ ability to mitigate flooding and erosion, causing significant ecological and economic losses. Additionally, Holocene tectonic subsidence and suppressed shell material production, exacerbated by rising sea levels, contribute to erosion and alter the spits’ contours, landscape outlines, and vegetation cover, driven by both anthropogenic and natural factors.

Shortly after the Russian full-scale invasion in 2022, the ATS were occupied. This led to catastrophic environmental consequences classified as ecocide. Military activities, including construction of fortifications, shelling, fires, use of landmines, explosives and chemical contamination, have severely impacted the spits and their vegetation, posing increased ecological risks. Existing infrastructure, including recreational facilities, suffered destruction, affecting essential services. Transitioning from ATS destruction to recovery depends on ending the war, halting destructive factors, and formulating restoration strategies, which can facilitate the gradual recovery of the spits.

Therefore, our monitoring efforts aim to identify environmental changes in the ATS region, including geomorphological transformations and vegetation alterations, in response to climate change and other anthropogenic influences. This helps prevent undesirable consequences of inadequate interventions. The inquiries prompted by this research could be better addressed in forthcoming monitoring endeavors, necessitating field surveys on ATS, which are currently impossible due to the ongoing war and occupation.

## Conclusions

1. The vegetation of the ATS has undergone significant changes in a relatively short period, driven by ongoing global sea level rise and climatic shifts characterized by pronounced aridization, as well as anthropogenic influence, particularly intensified in recent decades.

2. Morphodynamic characteristics of ATSP reveal dynamic coastal processes predominantly influenced by sea level fluctuations, wind-wave regime alterations, changes in sea salinity and variations in biogenic material volumes, emphasizing the role of inundation. Over time, notable alterations on ATSP, such as shifts in land area, littoral zone washouts, sediment deposition, dynamic movement or separation of ATSP tips, intensified erosion, and increased landslides, underscore the dynamic nature of these coastal environments.

3. Our research results underscore significant vegetation changes in ATSP over the last 95 years. The increase in the area of halophytic meadow and marsh plant communities occurred alongside a certain decrease in their productivity, while the reduction in sandy-steppe plant community areas was accompanied by an increase in their productivity. This corresponds to the prevailing inundation trend. Specifically, the rise in NDVI and the decline in NDWI, amid increasing annual air temperatures and reduced annual precipitation, signify the maintenance of adequate water supply to plants even in the face of diminished rainfall and a reduction in the expanse of inland water bodies. These shifts, driven by factors like sea level rise, climate change, tectonic subsidence and human activities, are expected to persist, underscoring the crucial need for ongoing monitoring efforts to comprehend and address the dynamics of ATS ecosystems, especially in light of substantial projected sea level rise by 2050.

4. The ongoing anthropogenic transformation of the ATS and their vegetation aligns with local human-induced alterations in the ecosystems of the Sea of Azov and the ATS, as well as global climate changes. This reaffirms our forecast made two decades ago and is expected to escalate in the future, particularly in light of potential global temperature change scenarios and semi-empirical scenarios for Ukraine, projecting a continued increase in the average surface temperature in the study region until 2050.

5. Remote sensing tools offer valuable insights into vegetation dynamics, serving as a complementary tool for validating field data and monitoring when field research is limited. They are particularly useful for identifying general trends in the state of plant communities and verifying forecasts for changes in vegetation cover.

## Supplemental Information

10.7717/peerj.18369/supp-1 Supplemental Information 1 Different Temporal Climate Indicators of NA

10.7717/peerj.18369/supp-2 Supplemental Information 2 Retrospective vegetation maps

10.7717/peerj.18369/supp-3 Supplemental Information 3 ERS tools

10.7717/peerj.18369/supp-4 Supplemental Information 4 Features of morphodynamics on each spit

10.7717/peerj.18369/supp-5 Supplemental Information 5 Potential drivers of ATSP vegetation changes

10.7717/peerj.18369/supp-6 Supplemental Information 6 Location of the meteorological stations of NA and sources

10.7717/peerj.18369/supp-7 Supplemental Information 7 Characteristics of satellite image sources, time period, and number of images used for the analysis

## Additional Information and Declarations

### Data Availability

The following information was supplied regarding data availability: The data from Climate Explorer is available at http://climexp.knmi.nl/selectstation.cgi?id=someone@somewhere. CGO: Central Geophysical Observatory of empirical data. http://cgo-sreznevskyi.kyiv.ua/index.php?lang=en&fn=u_klimat&f=ukraine&p=1
The data from NASA Global Climate Change is available at: https://climate.nasa.gov. The data from NASA Sea Level Change is available at: https://sealevel.nasa.gov. The data from Open Data–Server is available at: https://opendata.dwd.de/climate_environment.
