# Supplementary material for: Azov-type spits: long-term monitoring of morphodynamics and vegetation in response to changing environment
Source: PeerJ. 2025 Apr 16;13:e18369. doi: 10.7717/peerj.18369 (PMC12009029; doi:10.7717/peerj.18369)
Supplement: Supplementary file 1 [file peerj-13-18369-s001.odt]

Supplement 1. Different Temporal Climate Indicators of NA

The analysis of the causes of vegetation cover changes in Azov–type spits was partially based on a set of statistically processed meteorological data obtained from several meteorological stations in the south of Ukraine for the period 1900–2021. This data includes average annual and average values of surface temperature and precipitation.

In this study, empirical data from meteorological stations located on the territory NA were used (Table S1). The dataset included averaged monthly surface air temperature (oC per month) and precipitation (mm per month), spanning the period from 1900 to 2021 for stations, Henichesk, Mariupol, Melitopol (Open Data–Server, 2022; CGO: Central Geophysical Observatory of empirical data, 2021; Climate Explorer, 2023). The study also incorporated climatic norms of surface air temperature (Tnrm) and precipitation (Pnrm) for stations Berdiansk, Botieve, Henichesk, Mariupol, Melitopol for the periods of 1961–1990 (The Climate Cadastre of Ukraine, 2005) and 1991–2021 (CGO: Central Geophysical Observatory of empirical data, 2021). It is important to note that these meteorological stations in NA have been located in an occupied zone since February 2022, and it is likely that a majority of them have been destroyed. Therefore, empirical data from meteorological observations at these stations are not available from the Central Geophysical Observatory of Ukraine (CGO).

In this research, we used the period from 1900–2021 to illustrate long–term trends in surface temperature and precipitation and compared these trends with more recent changes from 1991–2020. The climatic norms for temperature and precipitation for the period 1991–2020 were calculated and compared to norms for the period 1961–1990 obtained from another source. To assess the trends in the seasonal progression of temperature and precipitation, we calculated the coefficients of linear trends (Ttrn and Ptrn) and standard deviation (σ) for each month at each meteorological station and then averaged these over the territory under study. The trends in meteorological parameter changes were approximated using linear regression. We evaluated the statistical significance of these trends using the nonparametric Mann–Kendall test, which is widely used for detecting trends in time–series data (Storch, Zwiers, 1984). A 95% significance level, a commonly chosen threshold in statistics, was used. The trend values for the long series over the period 1900–2021 were normalized by a factor of 100, and those for the climate normal over the period 1991–2020 were normalized by a factor of 10. The results were based on data processed according to the methods of statistical analysis of meteorological information. The statistical analyses and graphical designs were performed in MS Excel and XLSTAT software.
