# Supplementary material for: Azov-type spits: long-term monitoring of morphodynamics and vegetation in response to changing environment
Source: PeerJ. 2025 Apr 16;13:e18369. doi: 10.7717/peerj.18369 (PMC12009029; doi:10.7717/peerj.18369)
Supplement: Supplementary file 2 [file peerj-13-18369-s002.odt]

Supplement 2. Retrospective vegetation maps

The initial state of vegetation on the ATS was assessed using vegetation maps published by Ukrainian scientists at the onset of the 20th century. The majority of them conducted comprehensive studies on the plant life of the spits as part of the "Nadmorski zapovidnyky" (Seaside Reserves in English), recognized as one of the world's pioneering protected areas. Specifically, in this study we used S. Postryhan's maps from 1927-1929 for Kryva, Bilosaraiska, and Berdianska spits (Postryhan, S.A., 1939) and P. Popovych's map from 1934 for Obytichna spit (Popovych, 1936), executed at scales of 1:30000 - 1:90000 (Figure S1).

The large-scale maps, we developed in the 1990s, enabled us to extend research conducted in the 1920s and 1930s, forming the basis for a continuous monitoring series (Figure S2). Our study from the 1996 to 1999 within the territories of ATSP relied on detailed route-based field investigations, accompanied by large-scale cartographic surveys (1:10000 - 1:17000 scale) and 629 relevés. The field cartographic survey was conducted using a route-oriented traverse method, employing the parallel transect method with intervals of 70-200 m, along with ecological profiling. The cartographic basis for these maps comprised various materials, including aerial photographs, topographic maps of different scales, land use plans and forest management plans. Cartometric calculations for determining the areas of different map units were carried out manually using a planimeter. The thematic similarity of the compared maps from 1927-1934 and 1996-1999, despite different accuracy and reliability, allowed us to gain a comprehensive understanding of the spatial structure, organization, dynamics, and quantitative assessment of changes in the vegetation cover of the spits. The entire set of vegetation maps can be used to consider ATSP as special stationary polygons for monitoring natural processes in the NA region. These maps were initially published in O. Tyshchenko's monograph in 2006, accompanied by detailed legends. In the current study, they are represented as schematic illustrations in Figure S1 (from 1927-1934) and Figure S2 (from 1996-1999) to provide a general overview. These illustrations were slightly modified to improve clarity and perception: colors were added to dashed map units, and the longest spits – Berdianska and Obytichna on the 1996-1999 maps – were divided into sections. The names of vegetation syntaxa in the text are given according to the "Prodrome of the vegetation of Ukraine" (Dubyna et al., 2019).
