# Supplementary material for: Azov-type spits: long-term monitoring of morphodynamics and vegetation in response to changing environment
Source: PeerJ. 2025 Apr 16;13:e18369. doi: 10.7717/peerj.18369 (PMC12009029; doi:10.7717/peerj.18369)
Supplement: Supplementary file 3 [file peerj-13-18369-s003.odt]

Supplement 3. ERS tools

One of the aims of our research was to assess the feasibility of using ERS tools and satellite image databases to analyze morphological changes in ATSP and examine long-term alterations in their vegetation cover. This involved considering specific climatic, hydrological, and soil indicators influencing vegetation and verifying hypotheses concerning the consequences of global climate change on changes in vegetation.

This analysis was conducted on the EO Browser web platform ([https://apps.sentinel-hub.com/eo-browser/](https://apps.sentinel-hub.com/eo-browser/)) using accessible collections of satellite images and Sentinel Hub tools, utilizing several normalized indices for conducting in-depth research on vegetation, moisture content, water bodies, and ground temperatures: NDVI (Normalized Difference Vegetation Index), NDWI (Normalized Difference Water Index), NDMI (Normalized Difference Moisture Index)  and Thermal. 

NDVI is a dimensionless index that serves as a reliable indicator of vegetation cover productivity. It exhibits correlations with essential parameters, including chlorophyll content, leaf area, vegetation density, and is closely linked to productivity, soil moisture, evaporation, precipitation, and snow cover.

NDWI is a remote sensing derived index estimating the variations related to water content in water bodies. It relies on the green and near-infrared bands to emphasize water bodies, making it particularly suitable for water body mapping, where values exceeding 0.5 indicate the presence of water bodies.

NDMI is a geoinformation index, instrumental in determining moisture content within vegetation cover and monitoring drought conditions. This index quantifies changes in leaf water content by leveraging the reflectance coefficients of near-infrared (NIR) and shortwave infrared (SWIR) bands.

Thermal is an index used in remote measurement of surface temperature. The thermal visualization, based on TM Band 6, operates in the TIR (thermal infrared) radiation at a central wavelength of 11040 nm.  It delivers surface temperature data at a 120-meter resolution, resampled to 30 meters.

To analyze the data, the following satellite image sources were used: Landsat 1-5, Landsat 4-5, MODIS, and Sentinel-2,covering the period from 1975 to 2022 (Table S2).

The boundaries of ATSP for a long-term analysis of changes in each of the indices were determined from high-quality satellite images for each of the spits and stored in .json format. For the analysis of changes in NDVI and NDMI, areas encompassing large water bodies, urban areas, and agricultural lands were excluded from the study boundaries. The NDWI analysis sites covered large water bodies within the spits. The boundaries of sites dominated by specific types of vegetation were overlaid onto satellite images using the contours from original 1990s large-scale vegetation maps.

To evaluate long-term morphological changes in ATSP, we conducted measurements and comparisons of contours and the area of the visible land portion of the ATSP using high-resolution satellite images. Sentinel Hub tools allow for the measurement of closed-contour sites areas. Images with NDWI visualization were found to be the most suitable for this comparison, as they provide a clear distinction between land and water boundaries. We used NDWI to discriminate between water and land with a threshold value above 0.5. Since ATSP experience significant seasonal fluctuations in water levels (due to inflow phenomena and storms) for most of the year, we used images from the "most stable" period for the spits - August in 1976-1978 (Landsat 1-5, NDWI) and in 2022 (Sentinel-2, NDWI). Additionally, for comparison, we used images from intermediate periods in the 1980s and 1990s.

To obtain reliable statistical information on the dynamics of index parameter changes, we employed automatic image filtering based on cloud cover (Sentinel-2 - up to 10%) or manually selected images with low cloud cover and without image defects (Landsat 1–5, Landsat 4–5). For MODIS visualizations, images were filtered through visual analysis to identify and exclude those with atypical index values and deviations. 

For analysis, we used images from the vegetation period, considering image availability in the following intervals: June-August (NDVI Landsat 1–5, Landsat 4–5); April-August (NDVI MODIS); April-September (NDVI, NDMI Sentinel-2); May-September (Thermal Landsat 4–5); August (NDWI Landsat 1–5, Landsat 4–5, MODIS). The selection of periods for analyzing the multi-year dynamics of NDVI was determined by the availability of high-quality images during the growing season. Specifically, the number of high-quality Landsat images available for April, May, and September in certain years was insufficient for a reliable multi-year comparative analysis. Consequently, this led to a differentiated selection of periods, with images analyzed separately for each satellite. Using this approach, we did not compare generalized NDVI indicators (e.g., monthly or annual averages) across images from different satellites. Instead, we focused on the dynamics of index changes for each satellite  separately within specific time interval. Median values of index parameters were used. The results of the image visualizations obtained using Sentinel Hub tools in the form of statistical information were exported to a .csv file. Subsequently, the data was reformatted into .xlsx format, and trends in parameter changes were approximated using linear regression analysis. Statistical analyses and graphical designs were performed using LibreOffice Calc and MS Excel software. We determined the average values of the parameters for each month or year, constructed graphs illustrating long-term changes, added trendlines, calculated the scales of changes based on linear regression trends (in %), and calculated cumulative chain rates of change (in %).
