# Supplementary material for: Azov-type spits: long-term monitoring of morphodynamics and vegetation in response to changing environment
Source: PeerJ. 2025 Apr 16;13:e18369. doi: 10.7717/peerj.18369 (PMC12009029; doi:10.7717/peerj.18369)
Supplement: Supplementary file 4 [file peerj-13-18369-s004.odt]

Supplement 4. Features of morphodynamics on each spit

A typical Azov-type spit consists of three parts which illustrate the morphogenetic division of its body from the landward to seaward (Figure S3) - the base of the spit, located near the elevated coastal slope, the middle part, and the terminal end, historically known as "dzendzyk", where the most recent deposits are formed (Zenkovich, 1958; Kaplin et al., 1991; Tyshchenko, 2006). In this scheme arrows indicating the main direction of currents, wave activity, and intense terrigenous material transport along the spit's coastline, while the wind rose provides information about the prevailing wind directions in the region. The longitudinal zones (A, B, and C) represent distinct sections of the spit from the shoreline towards the sea. The Frontal (littoral) zone (A) experiences the direct impact of waves and storms, leading to the formation of relatively high littoral ridges. The Medial zone (B) plays a crucial role in the overall spit formation and is less susceptible to destructive sea storms. The Rear (backshore) zone (C) shares similarities with the Frontal zone but lacks intense and prevailing destructive storm impacts and accumulations of coarse terrigenous material. The transverse zones (1, 2, and 3) refer to areas across the spit's width. The Proximal (proximate to the mainland) zone (1) experiences the dominant influence of continental processes, including the influence of underground freshwater and aeolian activities, resulting in the formation of inland brackish lagoons and the prevalence of halophyte-meadow, halophyte, and estuary marsh plant communities. The Middle (central) zone (2) is more dependent on the sea's influence and is characterized by the presence of inland brackish lagoons, halophyte meadows, saline psammophytic steppes interspersed with strips of hydrophilic plant communities. The terminal (end) zone (3) is highly influenced by the sea and dominated by specific halopsammophytic plant communities. The southern and most dynamic tip of the spit is apparent in various geographical labels within the NA region, signifying the extremities of coastal spits or small islets that are shaped by the dynamic characteristics of the spit's tips, continually influenced by shifts and erosion.

Features of the morphodynamics of Kryva spit. When comparing the spit's area in 1998, approximately 1800 hectares, to its earlier state as mapped by S. Postryhan in 1927-29, around 2200 hectares, a significant overall reduction of about 18% becomes evident. Over a 70-year period, significant changes in the spit's outlines are primarily attributed to the elongation of its southern part, where the former island area has merged with the spit's dzendzyk, and the reduction of its 'body' area due to erosion on the eastern coast and weak accumulative processes on the western coast. The results of comparing the area and structure of the spit using satellite imaginary data from 1975 to 2022 show that the overall area of the spit has remained relatively unchanged during this time frame. However, specific parts of the spit have undergone alterations. In particular, the isthmus connecting the main part of the spit to the dzendzyk has narrowed by approximately 100-150 meters and has shifted slightly in a northwestern direction. Additionally, sediment deposition has resulted in accretion of up to 150 meters in the northwestern mainland area of the spit.

Features of the morphodynamics of Bilosaraiska spit. The main morphodynamic changes of Bilosaraiska spit from 1927-29 to 1998 included a nearly 5% reduction in total area (from approximately 3,900 hectares to around 3,750 hectares). Embankments were constructed to link sandy dunes and a small island near the southwest coast to the spit, creating a fish-breeding area, which was later abandoned due to declining fishing activities in the Sea of Azov. Inland lakes in the central part of the spit expanded over fourfold, increasing from 3% to nearly 14% of the spit's total area, and took on elongated shapes parallel to the western shore of the spit. The analysis of satellite image data from the period of 1975-2022 revealed that the spit's area increased by 0.3% during this timeframe. The tip of the spit was extended by 200-300 meters and curved in a northern direction. There was minor erosion in the central southeastern part of the spit and several small islets with Phragmito-Magnocaricetea plant communities have already submerged.

Features of the morphodynamics of Berdianska spit. From 1929 to 1998, Berdianska spit's total area decreased by nearly 18% (from 4000 hectares to almost 3300 hectares). By 1998, increased waterlogging was evident. Erosion of the dzendzyk led to Malyi Dzendzyk island's formation. The Great Bay started filling rapidly with sediments, giving rise to low-lying reed-covered islets and peninsulas with small interconnected lakes. Lakes in the northeastern part, occupying nearly 12% of the area in 1929, evolved into interconnected water bodies covering 18% by 1998. In the northwestern part (within Berdiansk city), the groundwater level rose. Human impact, driven by the development of recreational infrastructure and city expansion southwards along Berdiansk Bay's coast, increased by 9% from 1929 to 1998, covering 47% of the spit's total area (up from 38% in 1929). Satellite image comparisons from 1975 to 2022 show a 1.2% decrease in the area of Berdianska spit, notably due to coastal erosion in the central southeastern part, resulting in a width reduction of 50-100 meters. Malyi Dzendzyk Island slightly expanded southeastward. Accretion of 50-100 meters occurred in the southeastern tip and northwestern part of the spit, with the tip curving northward.

Features of the morphodynamics of Obytichna spit. During 1934-1996, spatial changes on the spit were challenging to measure accurately due to excessive generalizations, overall schematization, and the absence of a linear scale on P. Popovych's map. Likely, the author lacked precise cartographic data, possibly distorted during the challenging 1930s under the former Soviet regime. His map shows significant discrepancies in representing natural features like mainland contours and nearby lakes. Some differences can be attributed to natural changes in the spit's outlines. Comparing with a 1996 map, the spit's total length in 1934 was smaller by 200-300 meters, and its terrestrial area decreased by almost 22% (from around 3200 hectares in 1934 to about 2500 hectares in 1996). Moreover, there were fewer lagoons in the near-mainland part of the spit, and its overall elevation was slightly higher than in 1996. From 1975 to 2022, there was a 1.5% decrease in the area of Obytichna spit. Coastal erosion in the central southeastern part reduced the spit's width by 50-100 meters, with storms periodically affecting the narrowest section. Accretion widened the southern and southwestern tips by 200-300 meters, and changes in the dzendzyk's shape led to its separation, now an island.
