# Supplementary material for: Azov-type spits: long-term monitoring of morphodynamics and vegetation in response to changing environment
Source: PeerJ. 2025 Apr 16;13:e18369. doi: 10.7717/peerj.18369 (PMC12009029; doi:10.7717/peerj.18369)
Supplement: Supplementary file 5 [file peerj-13-18369-s005.odt]

Supplement 5. Potential drivers of ATSP vegetation changes

A detailed analysis of our assumptions regarding the potential drivers of the observed vegetation changes on ATSP is presented focusing on the primary types of plant communities.

The littoral zones of ATSP can be considered as indicators of the balance of sedimentation by coastal currents. Until recently, the accumulation processes ensured a balance or an excess of forming material for the spits. We attribute the nearly twofold reduction in the area of littoral zones and the associated littoral vegetation on ATSP to the weakening of accumulation processes primarily caused by anthropogenic factors, as elaborated upon in Section 4.2.

Sand-steppe plant communities, prevalent in the highest parts of ATSP near the coastal zone, suffered early impacts from human development. The decline in psammophytic-steppe plant communities on ATSP was driven by construction expansion on spits, beginning from the highest coastal areas. Extensive afforestation efforts aimed at recreational areas, living conditions, and coastal erosion and deflation protection contributed to their destruction. Consequently, psammophytic steppes on the spits were destroyed, displacing many waterfowl to alternative habitats. Their overall area notably decreased sixfold due to these developments. These communities, serving as nesting sites for waterfowl, now face increased disturbance on small islets and sandy dunes.

Small patches of zonal steppes (Festuco-Brometea) on the continental slopes adjacent to the ATSP and on the Stepok loess island, embedded within the Fedotova spit, have been significantly impacted by agricultural pressure, construction, plowing and the establishment of quarries for soil extraction. It is evident that these areas will not be able to return to their original state as typical zonal steppes in the near future.

The decline in areas covered by halophytic plant communities primarily occurred in former lowland saline areas, such as coastal strips surrounding shallow coastal lakes and depressions forming on the bottom of drying estuaries. This decline could only occur if the spits were inundated due to one or a combination of several processes, further discussed in section 4.2. This reduction is accompanied by a significant decrease in the accumulation of mollusk shells, a primary material for the formation of the spits. With high salt content persisting in all soils, groundwater solutions, water bodies, and substrates on the spits, these areas were transformed into salt marshes and halophytic meadows in most cases.

The expansion of inland water bodies on ATSP, observed through a comparison of vegetation maps from the 1920s to the 1990s, likely resulted from various factors, including climate changes, sea level fluctuations, declining spits elevation, rising groundwater levels, intensified lagoon hydrological fluctuations, and disruptions to natural development mechanisms of ATSP. Analysis of NDWI data revealed an inconsistent trend in expanding water body areas until the 1990s. In the 1970s, the sizes of internal water bodies on the ATSP were larger than in the 1990s, and cumulatively from 1975 to 2022, their areas gradually decreased. This trend aligns with the aridization trend in NA in recent decades, characterized by reduced average annual precipitation amid increasing average annual air temperatures. It is hypothesized that the soil moisture deficit, likely resulting from prolonged increases in average annual air temperatures and decreased precipitation, does not adversely affect major plant communities, as evidenced by the increasing vegetation index trend for most types of plant communities on ATSP. The increase in evaporation and the reduction in the area of internal water bodies, attributed to aridization, are likely compensated for by inundation, rising groundwater levels and vegetation overgrowth on shoaling areas. Evidence from satellite image data supports subsidence and spits inundation, with rising NDVI and declining NDWI suggesting sufficient water supply to plants despite decreased rainfall and water body area. Additionally, the increasing NDMI, particularly notable in 2018-2019, further supports this trend.
