# Supplementary material for: Azov-type spits: long-term monitoring of morphodynamics and vegetation in response to changing environment
Source: PeerJ. 2025 Apr 16;13:e18369. doi: 10.7717/peerj.18369 (PMC12009029; doi:10.7717/peerj.18369)
Supplement: Supplementary file 6 [file peerj-13-18369-s006.odt]

TABLE S1. Location of the meteorological stations of NA and sources.

| Station | Altitude, m | Latitude, grad | Longitude, grad | Periods/Source |
| --- | --- | --- | --- | --- |
| 1900–2021 | 1961–1990 | 1991–2020 |
| Berdiansk | 2 | 46.75 | 36.78 | (Open Data–Server, 2022; CGO: Central..., 2021; Climate Explorer, 2023) | (The Climate Cadastre..., 2005) | (CGO: Central..., 2021) |
| Botieve | 19 | 46.68 | 35.85 |
| Henichesk | 15 | 46.16 | 34.81 |
| Mariupol | 70 | 47.03 | 37.50 |
| Melitopol | 34 | 46.83 | 35.36 |
