# Supplementary material for: Azov-type spits: long-term monitoring of morphodynamics and vegetation in response to changing environment
Source: PeerJ. 2025 Apr 16;13:e18369. doi: 10.7717/peerj.18369 (PMC12009029; doi:10.7717/peerj.18369)
Supplement: Supplementary file 7 [file peerj-13-18369-s007.odt]

TABLE S2. Characteristics of satellite image sources, time period, and number of images used for the analysis 

| Satellite image source | Sensors | Spatial resolution, m | Image acquisition frequency, days | Years | Number of images |
| --- | --- | --- | --- | --- | --- |
| Landsat 1–5 | MSS L1 | 60 | 16–18 | 1975–1993 | 599 |
| Landsat 4–5 | TM, TM Band 6 | 30 | 16 | 1984–2011 | 575 |
| MODIS | Moderate Resolution Imaging Spectroradiometer | 250, 500 | 1-2 | 2000-2022 | 16723 |
| Sentinel-2 | L2A | 10, 20, 60 | 5 | 2017–2022 | 778 |
